# Stop burning out and breaking the healers: burnout, depression, and the urgent need to humanize medical school in the Latin American context

**DOI:** 10.3389/fmed.2026.1789972

**Published:** 2026-03-12

**Authors:** Jose E. Leon-Rojas

**Affiliations:** 1 Grupo de Investigación Bienestar, Salud y Sociedad, Escuela de Psicología y Educación, Universidad de Las Américas, Quito, Ecuador; 2 Grupo de Investigación en Humanidades Médicas y de la Salud (HUMS), Escuela de Medicina, Universidad de las Américas, Quito, Ecuador

**Keywords:** abuse, depersonalization, emotional exhaustion, harassment, mistreatment, reduced personal accomplishment

## 1 Introduction

Medical students enter training driven by a passion to heal, yet all too often the journey through medical school leaves them burnt out, depressed, and demoralized. In recent years, the alarming prevalence of burnout and mental health struggles among medical students has reached crisis levels worldwide; studies estimate that roughly half of all medical students experience burnout before they even graduate. A 2,018 systematic review and meta-analysis that included 17,431 medical students reported a pooled burnout prevalence of 44.2% (95%CI, 33.4–55.0); the main reported causes were emotional exhaustion, depersonalization, and the pursue of a personal accomplishment (1). This meta-analysis provides foundational data on global depression prevalence among medical students, but it was conducted before the COVID-19 pandemic and may not fully capture more recent shifts in mental health trends (1). Another pre-pandemic study reported that one in three medical students have had depression with less than one in ten seeking proper help (2). Subsequent studies and meta-analyses conducted during and after the COVID-19 era have reported high rates of depression among medical students, often exceeding pre-pandemic estimates and highlighting persistent stressors associated with training and pandemic-related disruptions (3, 4). For example, meta-analytic evidence suggests that pooled prevalence of depression during the COVID-19 pandemic ranged from approximately 30 % to nearly 50 % depending on region and measurement approach (3). This quiet epidemic not only harms future physicians, but also threatens the quality of care they will provide to patients. Therefore, in this opinion article I would like to pose and confront an urgent question: If we continue to burn out our healers in training, who will be left to care for us?

For conceptual clarity, it is important to distinguish between related but non-equivalent terms used throughout this opinion article. Burnout refers to a work-related syndrome characterized by emotional exhaustion, depersonalization, and reduced personal accomplishment, whereas depression denotes a clinical or subclinical mood disorder with broader affective and cognitive features. In contrast, mistreatment, harassment, and abuse describe forms of inappropriate conduct within educational or clinical environments. Mistreatment encompasses a broad range of behaviors perceived as disrespectful or humiliating; harassment typically involves repeated, targeted behaviors often linked to protected characteristics or power asymmetries; and abuse refers to more severe or coercive actions that may be psychological, verbal, or physical. While these constructs are interrelated and may co-occur, they are conceptually distinct and play different roles as exposures or outcomes within health professions education.

## 2 A global epidemic of burnout and depression

Burnout in medical school is characterized by emotional exhaustion, depersonalization (cynicism), and a diminished sense of accomplishment (5). These symptoms, first noted in medical trainees decades ago, have only become more prevalent (6). Recent meta-analyses put the global prevalence of medical student burnout at around 37–45% on average, meaning nearly one in two students is affected; with some reporting prevalences as high as 88% (1, 7). This far outpaces burnout levels seen in many other high-stress fields (8). Equally concerning are the rates of depression and suicidal ideation among medical students. A comprehensive 2016 review in JAMA reported that 27.2% of medical students worldwide showed depression or depressive symptoms, a rate several times higher than that of their peers in the same age group; with 11% of them reporting suicidal ideation and only 15.7% seeking psychiatric aid (2). To put that in perspective, medical students' risk of depression is estimated to be 2.2–5.2 times higher than for individuals of similar age in the general population (2). In fact, some research indicates that medical students may suffer higher rates of depression, suicidal ideation, and feelings of low achievement than even resident physicians or young doctors; highlighting that medical school itself is a uniquely intense crucible of distress (9). This combination of burnout and mental health struggles has serious consequences: it impairs students' learning, affects their empathy toward patients, and has been linked to increased risk of medical errors and future career dissatisfaction (10–12). If half of our future doctors are burning out before they can wear the white coat as physicians, the downstream effects on healthcare quality and physician workforce stability are profound and worrisome.

## 3 Culture and pressure: why medical students are suffering

Why are medical students so burned out? The causes are multi-factorial, but a toxic culture of overwork and mistreatment is often at the center (11, 13, 14). A survey that included more than 500 third year medical students reported that medical students had had at least one incident of mistreatment by faculty (64%) or by residents in hospitals/clinics (76%); furthermore, 10% and 13% of students reported several mistreatments by faculty and residents, respectively (15). Beyond burnout, sustained exposure to mistreatment and chronic academic stress has been consistently associated with increased depressive symptomatology among medical students, including feelings of hopelessness, persistent low mood, and, in severe cases, suicidal ideation (3, 16, 17). Unlike burnout, which is conceptualized as a work-related syndrome, depression carries broader functional and long-term mental health implications, further underscoring the gravity of institutional neglect.

Medical training is notoriously demanding; long hours of study and clinical duties, high-stakes exams, fierce competition, and the ever-present weight of expectations from family and teachers alike (18–22). Students often find themselves sacrificing sleep, basic self-care, and personal relationships in order to meet relentless academic requirements (18–22). Over time this chronic stress and imbalance significantly affects their well-being. It's no surprise that by the end of the first year, many students report high emotional exhaustion (18–22). Beyond workload, the cultural norms of medical education have historically downplayed student wellness (23). The field has long glorified toughness and stamina, the ability to “push through” fatigue and emotional strain, as a badge of honor or as a rite of passage (23). In some environments, taking time for oneself or admitting to stress is stigmatized as a sign of weakness (24, 25); my country is one of these environments. This mentality pressures students to suffer in silence rather than seek help, fueling cycles of anxiety and burnout. Indeed, researchers note that many medical students hide their struggles “for fear of being stigmatized,” which can lead to underreporting of burnout and delay in getting support (24–26).

Alarmingly, mistreatment and bullying remain common experiences for medical students, further contributing to burnout. From public belittlement on ward rounds to sexist or racist remarks, harassment in the training environment adds an extra layer of psychological harm (14, 27). Surveys in the United States have found that the majority of medical students experience at least one incident of mistreatment during their training. Repeated abuse is not rare; in one multicenter US. study, about 11–13% of third-year students reported being mistreated “numerous times” by faculty or residents (15). Crucially, those who endured frequent mistreatment had dramatically higher burnout rates (approximately 57%) compared to those who did not (32%) (15). This data underscores what should be obvious, a culture of harassment and humiliation is fundamentally incompatible with student wellbeing. Yet despite decades of calls for change, mistreatment persists in medical schools worldwide, especially in countries were wellbeing and mental health are still taboo topics or are seen as non-important ramblings of the youth that “likes to complain”.

In my view, the consistency of these findings across countries and institutional settings makes it difficult to interpret burnout and depression as isolated or purely personal phenomena. Rather, they reflect systemic features of how medical education is organized and legitimized. It showcases the daily struggles of medical students as well as the mistakes we, as educators and trainers, need to face and work to correct.

## 4 Latin America: shared struggles, unique context

Burnout and mistreatment of medical trainees are global phenomena, but it is important to consider regional contexts. In Latin America, medical students face many of the same stressors seen elsewhere, often compounded by resource constraints and cultural norms. Notably, some evidence suggests a paradox in reported burnout rates versus experiences of mistreatment in the region. A 2019 international meta-analysis found that the highest burnout prevalence among medical students was observed in the Middle East, while the lowest was in South America (1). For example, one Brazilian meta-review in 2017 reported a burnout prevalence of only 13.1% among medical students (28); far lower than estimates from North America or Europe. On the surface, this could imply South American students are faring better. However, such numbers may be misleading or reflect differences in measurement. In the same Brazilian analysis, nearly 30.6% of students screened positive for depression, suggesting significant distress persists (28). Some experts speculate that burnout might be underreported or defined differently in Latin American studies, or that strong family/social support networks could be buffering stress even as academic pressures mount. However, another systematic review focused solely on Latin American medical students reported prevalences of burnout syndrome ranging from 4.3% to 43.9% (29).

On the other hand, evidence of medical student mistreatment in Latin America is disturbingly high. In Brazil, a cross-sectional study in a public medical school found that 30.1% of students recurrent mistreatment, with 92.3% reporting at least one event of mistreatment during their training years and 64.2% reported being exposed to severe mistreatment (16). Even worse, a 2024 descriptive cross-sectional study done in Ecuadorian medical students reported that 96.2% of participants had experienced at least one episode of mistreatment and only 72.5% recognized it as such (18); meaning that, in my country and, I believe, in many more Latin American countries than those reported in the literature, abuse and mistreatment is so pervasive that students perceive it as “normal” and have difficulty identifying it. The abuse was predominantly verbal and psychological; cruel teasing, humiliation, and harsh scolding, most often perpetrated by faculty members in positions of power in 87.9% of cases (18). As students advanced in training, incidents of mistreatment only increased, peaking during the clinical internship years. The impacts were severe; 90% of affected students reported feeling “diminished and depressed” as a result of the abuse, but the rate of non-reporting such abuse is worrisomely high, as 90.6% of students decided to not speak due to fear of retaliation and institutional ineffectiveness (18). Distressingly, studies from other Latin American countries echo similar of mistreatment. Surveys in Chile, Peru, Mexico, and elsewhere have documented prevalence of student abuse exceeding 90% of those surveyed at some point in training (17, 30–32). In other words, virtually all students in certain institutions reported experiencing belittlement, harassment or other forms of academic violence. These figures contradict and disprove any notion that Latin America's lower reported burnout in global reviews means students are sailing through unscathed; on the contrary, many are enduring a toxic training environment, even if they don't label the outcome as burnout. The institutional failure to address mistreatment does not merely contribute to burnout; it may also exacerbate or precipitate depressive disorders, particularly in environments where stigma and fear of retaliation discourage help-seeking. This dual impact strengthens the ethical imperative for structural reform.

Moreover, evidence from Bolivia, Colombia and Argentina further illustrates that burnout, depressive symptomatology, and mistreatment during medical training are not isolated national phenomena but part of broader regional patterns (33, 34). Studies conducted in Colombian medical schools have reported high prevalence rates of burnout and clinically significant depressive symptoms among medical students, with emotional exhaustion and hopelessness frequently associated with academic overload and perceived lack of institutional support (35, 36). Importantly, these findings mirror patterns observed elsewhere in the region, where psychological distress is closely intertwined with structural and cultural aspects of medical training rather than solely individual vulnerability. Similarly, research from Argentina has documented mistreatment and hierarchical abuse during both undergraduate medical education and residency training (37, 38). Reports of humiliation, verbal aggression, and normalization of harsh supervisory practices suggest that deeply embedded academic hierarchies continue to shape training environments in ways that may contribute to burnout and depressive outcomes. These findings reinforce the argument that the problem is not confined to a limited subset of countries but reflects recurring institutional dynamics across diverse Latin American contexts.

In many Latin American medical schools and teaching hospitals, institutional and organizational structures play a central role in shaping trainee experiences. Academic hierarchies are often highly vertical, with strong asymmetries of power between students, residents, faculty, and senior clinicians. In such environments, mistreatment may be normalized as part of professional socialization, while fear of retaliation can discourage reporting. Formal mechanisms for addressing complaints, where they exist, are frequently perceived as opaque, ineffective, or insufficiently independent, limiting trust in institutional accountability. These structural features may contribute to the persistence of mistreatment and to the under-recognition of its psychological consequences. It is also important to recognize that Latin America encompasses diverse educational systems, regulatory frameworks, and institutional cultures. The organization of medical training, availability of mental health resources, and enforcement of professional standards vary considerably across countries, as well as between public and private institutions. While some universities have begun to implement wellness initiatives and reporting pathways, others lack formalized policies or the resources required for sustained intervention. Consequently, the patterns described here should be understood as uneven and context-dependent rather than uniform across the region.

It is worth noting that Latin America's medical education culture has traditionally been quite hierarchical. The professor or attending wields tremendous authority, and open critique of superiors is rare. In such settings, I believe that abusive teaching practices can become ingrained and perpetuated as a rite of passage or as a normality; “I suffered through it, so you must as well.” The lack of robust institutional policies against mistreatment until recently has allowed this cycle to continue. Furthermore, resource limitations (high student-to-teacher ratios, limited support services) in some Latin American institutions can leave students feeling especially helpless and unsupported (39). The result is a perfect storm for burnout, even if not always formally measured, resulting in high stress, high abuse, and little relief. This region-specific context highlights that while the burnout crisis is global, solutions must be culturally tailored. Latin American medical schools may need to place extra focus on eradicating deeply rooted mistreatment and expanding mental health resources, alongside the general wellness strategies applicable everywhere. I'm certain that Latin America now faces a strategic opportunity: to move from documenting harm toward institutional reform grounded in accountability and transparency.

## 5 Power, accountability, and the failure of institutional responses

A critical and often underaddressed dimension of mistreatment in medical education is the role of institutional power and the persistent failure of accountability mechanisms. Although mistreatment of medical students, interns, and residents has been repeatedly documented and reported to academic and hospital leadership, meaningful consequences for perpetrators remain uncommon (12, 13, 21). In many institutions, particularly those with rigid hierarchies, abusive behaviors by senior faculty or supervisors are tacitly tolerated, minimized, or actively concealed to protect institutional reputation or influential individuals (21, 25). This dynamic creates an environment in which reporting is perceived as futile or dangerous, reinforcing silence and normalizing abuse (13, 14). Existing reporting mechanisms frequently fail to deliver justice. Students and trainees often fear retaliation, negative evaluations, or damage to future career prospects, especially when the accused individual holds evaluative or supervisory authority (22–24). Moreover, complaint processes are commonly managed internally by the same structures that benefit from maintaining the status quo, creating conflicts of interest and undermining trust (21). As a result, many institutions prioritize risk management and reputational protection over ethical responsibility, allowing patterns of mistreatment to persist across cohorts (12, 13, 28).

While some medical schools have introduced wellness programs, counseling services, and peer-support initiatives, these measures primarily offer symptomatic relief and do not address the underlying power imbalances that enable abuse (2, 11). Ethical and professional accountability requires more than support services; it demands enforceable standards of conduct. Potential mechanisms include independent offices, anonymous reporting systems with external oversight, clear anti-retaliation protections, and the incorporation of teaching professionalism and trainee wellbeing metrics into faculty promotion and leadership evaluations. Evidence suggests that such measures are only effective when they are transparent, mandatory, and accompanied by genuine institutional commitment to enforcement (12, 13).

The persistence of mistreatment in medical education reflects not a lack of knowledge about the problem, but a failure of moral courage and governance. I believe that addressing this issue requires institutions to move beyond symbolic policies and confront the uncomfortable reality that excellence in clinical expertise does not excuse ethical misconduct. The central issue, therefore, is not whether mistreatment exists (the evidence makes that clear) but whether institutions are willing to confront the hierarchical structures that enable it. I argue that without structural accountability mechanisms, individual-level wellness interventions will remain insufficient. Only by realigning power, accountability, and professional values can medical education begin to dismantle cultures of abuse and replace them with environments that are both rigorous and humane.

## 6 Discussion: how to humanize medical training – recommendations

Several Latin American studies demonstrate both the prevalence of mistreatment and the institutional challenges in addressing it, suggesting the need for structured reporting mechanisms and support programs within medical schools (18, 33). High academic burnout rates documented in Bolivia have been used to justify the establishment of psychological support centers for students (34), and research from Chile highlights the importance of educational environments that promote psychological safety and wellbeing (40). Additionally, evidence from Peru and Mexico underscores substantial mental health concerns among medical students that would benefit from integrated support strategies (41). Furthermore, in other contexts, the appointment of independent ombudspersons or the establishment of professionalism committees with student representation has improved transparency and trust in reporting processes (42). Such models illustrate that accountability mechanisms can be institutionalized without undermining academic standards or clinical training quality.

To truly address this crisis, medical schools across the world must make a culture shift; from viewing student wellness as optional or secondary, to treating it as foundational to professional development. Humanizing medical education means recognizing that medical students are not just future doctors in training, but human beings with limits, emotions, and needs in the present. Therefore, below I provide several urgent steps and policy changes that could begin to turn the tide and benefit our students.

1) Promote a Supportive, Non-Toxic Learning Environment: The era of training by intimidation must end; schools should enforce zero-tolerance policies for harassment, belittlement, verbal violence, and discrimination. Faculty and residents need formal training in respectful supervision, feedback delivery, and recognition of student distress; in Latin America, medical school teachers often do not receive structured preparation in pedagogy or student psychology. However, training alone is insufficient. Institutions must structurally reinforce a respectful culture through transparent governance mechanisms, clear codes of conduct, independent reporting pathways, and enforceable consequences for violations. Respect should not depend on individual goodwill but be embedded in institutional policy and leadership accountability. Although hierarchies are intrinsic to medical training, they need not be authoritarian. A horizontal learning climate, where questioning is permitted, dialogue is encouraged, and students are treated as legitimate members of the professional community, is compatible with academic rigor and clinical excellence. Authority should facilitate mentorship and responsibility, not humiliation or fear. Students who are treated with dignity, fairness, and psychological safety learn more effectively and are more likely to develop into ethical and resilient physicians.
2) Implement Wellness and Mental Health Support Services: Every medical school should provide easily accessible, confidential mental health services for students. This includes on-campus counselors or therapists, stress management and resilience workshops, and peer support groups. Importantly, schools must destigmatize their use; leadership should openly encourage students to seek help just as they would encourage academic tutoring. Proactive measures like wellness check-ins or required counseling sessions each year (with no punitive consequences) can help normalize caring for one's mental health.
3) Allow Time Off and Encourage Self-Care: Medical students urgently need protected time for rest, health care appointments, and personal life. Rigorous training should not translate into continuous, unbuffered exposure to academic and clinical demands. Internationally, some medical schools have begun incorporating structured solutions; for example, the introduction of “bridge weeks” between clerkships in certain institutions allows students time for licensing examinations, medical appointments, and recovery before transitioning into new rotations. These initiatives demonstrate that curricular flexibility can coexist with high academic standards; Latin American institutions could adopt context-appropriate adaptations of similar models, even within resource-constrained environments, by redesigning rotation schedules, formally integrating wellness intervals, and ensuring flexible leave policies. Students facing physical or mental health challenges should be able to temporarily adjust workload or take protected leave without fear of academic penalty or reputational harm. Institutionalizing protected time communicates a clear message that sustainability and professional longevity are valued as much as endurance.
4) Reform Grading and Evaluation Systems: Hyper-competitive grading curves and constant high-stakes exams drive anxiety and cut into any sense of collegiality. Minimizing class rankings and honors distinctions can ease the pressure for students; additionally, recognition should be considered not only to the highest grade but also to those that significantly improve their grades as a way of incentivizing self-improvement rather than cut-throat competition. Evaluations should focus on competency and growth, not ruthless numeric comparisons. When students are not obsessing over grades, they are more likely to collaborate and support their peers, improving the learning climate.
5) Integrate Wellness into the Curriculum: Just as students learn anatomy or pharmacology, they should learn about burnout, resilience, and work-life balance as part of their training. Sessions on mindfulness, time management, and coping strategies can provide valuable tools. Initiatives like mentoring programs, where each student is paired with a faculty mentor to regularly discuss challenges, can also create a safety net. Some schools have introduced courses on physician wellness and reflective groups where students can openly share experiences. These are not “soft” add-ons; they are teaching the skills that help sustain a lifetime in medicine.

Above all, humanizing medical school requires a philosophical shift: students' wellbeing must be valued as much as their academic achievements. This is not just altruism, it is grounded in the reality that a healthier, happier trainee will become a more competent and compassionate physician. As Dr. Ariel Frajerman, who led a major burnout study, noted, the first step is “to consider the students as human being who can suffer,” and to build support structures accordingly (1). He also emphasizes reducing workload and exam burdens (for instance, eliminating unnecessary ranking systems) and improving the way faculty interact with students (1). Simply put, medical schools must care for their students, so that their students can learn to care for others.

## 7 Conclusion: time to care for the caregivers-in-training

This opinion piece advances a clear position: that the normalization of burnout and depression in medical training is ethically indefensible and institutionally avoidable. Certainly, burnout and depression in medical education is unlikely to be resolved through isolated or short-term interventions. It reflects a longstanding cultural problem embedded in the ways physicians are trained, shaped by decades of normalization and institutional inertia. Increasing evidence suggests that current approaches are insufficient, particularly when mental health concerns among medical students are treated as expected or inevitable aspects of professional formation. Accepting burnout, psychological distress, or despair as “normal” carries significant consequences, not only for individual trainees but also for the sustainability and ethical integrity of the medical profession. The consequences of inaction extend beyond individual wellbeing. Persistent exposure to mistreatment and unaddressed distress has been associated with attrition, long-term mental health morbidity, and diminished capacity for empathy and professional engagement. These outcomes represent a systemic risk to healthcare systems that rely on a resilient and compassionate workforce. At the same time, emerging evidence indicates that change is possible. Across different regions, students are increasingly voicing concerns, and a growing body of research on wellness in medical education is informing institutional responses. Several institutions have begun to implement structured wellness and support programs, with early reports suggesting improvements in learning environments and student satisfaction. In Latin America, where empirical data on mistreatment, burnout, and mental health in medical training remain comparatively limited, there is a timely opportunity to expand research efforts and inform context-specific reforms across both public and private universities. Collaborative, multinational research initiatives may play a critical role in identifying structural drivers of distress and in developing interventions that are responsive to regional realities.

Ultimately, addressing burnout in medical education is central to sustaining the core values of medicine. Medical students represent the future clinical workforce, and training environments that tolerate neglect or abuse risk undermining professional development and patient care alike. Importantly, many physicians have become excellent clinicians not through humiliation or intimidation, but through rigorous training guided by strong mentorship, ethical supervision, and environments that foster respect; these examples demonstrate that medical education does not need to rely on mistreatment to produce competence. The notion that mistreatment is a necessary or character-building component of training lacks empirical support and overlooks those whose careers were derailed by such experiences. Preventing burnout therefore requires a shift from endurance-based models of training toward educational cultures that prioritize wellbeing, professionalism, and ethical responsibility. In doing so, medical schools uphold a foundational principle of medicine: *primum non nocere* –first, do no harm, including within their own training systems.
